# The organization, weaknesses, and challenges of the control of thalidomide in Brazil: A review

**DOI:** 10.1371/journal.pntd.0008329

**Published:** 2020-08-06

**Authors:** Soraya Machado de Jesus, Rafael Santos Santana, Silvana Nair Leite

**Affiliations:** 1 Postgraduate Program in Pharmaceutical Sciences, University of Brasília, Brasília, Distrito Federal, Brazil; 2 Department of Pharmacy, University of Brasília, Brasília, Distrito Federal, Brazil; 3 Department of Pharmaceutical Sciences, Federal University of Santa Catarina, Florianópolis, Santa Catarina, Brazil; Johns Hopkins Bloomberg School of Public Health, UNITED STATES

## Abstract

The drug thalidomide has resurged in the world market under restrictive conditions for marketing and use. In Brazil, there are still cases of pregnant women using thalidomide even after the implementation of laws that regulate the control of use (Law No. 10.651/2003 and Collegiate Board Resolution No. 11/2011). The objective of this study was to discuss the control of thalidomide use in Brazil, based on a scoping review of the scientific literature, documents, and data from the Ministry of Health. A total of 51 studies and documents related to the following subthemes were selected: (1) organization of access and use of thalidomide in the health system; (2) epidemiological and population characteristics of people affected by leprosy; and (3) occurrence of pregnancy and cases of embryopathy with the use of thalidomide. The results showed that Brazil has no unified information database about thalidomide patients. Furthermore, there is inconsistency in the accreditation of public health centers that dispense this medicine, in a country that has a high consumption of thalidomide in the Unified Health System. A large part of this amount of dispensed medicine is intended for the treatment of erythema nodosum leprosum, mainly in the North, Northeast, and Central-West regions of the country, which are endemic for leprosy. This disease is the only one among the clinical indications of the medicine approved in Brazil that does not have a Clinical Protocol and Therapeutic Guidelines. The control of thalidomide use in Brazil presents historical regulatory failures. These are currently linked to the organization and structure of primary healthcare in the country, as well as to the lack of leadership of the Ministry of Health and National Health Surveillance Agency when it comes to managing the process of control of this use.

## Introduction

Thalidomide was discovered by Wilhelm-Kunz in 1953 in Germany, and it was synthesized by the Chemie Grünenthal lab in 1954. Testing in animals showed sedative and hypnotic effects and a lack of undesirable effects on the day after its consumption. Testing also showed the absence of a relevant lethality rate, even when high doses were used. In 1956, the lab extrapolated the results to humans and printed informative leaflets discriminating a series of therapeutic indications for thalidomide: irritability, lack of concentration, panic state, premature ejaculation, premenstrual syndrome, fear of being examined, functional disorders of the stomach and the gall bladder, febrile infectious diseases, light depression, anxiety, hyperthyroidism, and tuberculosis; such leaflets also advocated its use by pregnant women [1,2]. In the following year, Chemie Grünenthal promoted a variety of advertisement campaigns focusing on doctors and pharmacists around the world, and the medicine quickly reached the market in more than 46 countries, including Brazil, under various brand names [1,3,4].

Because of the great sales success, in 1958, a beverage company Distillers Biochemicals Ltd (DBCL) became a distributor of the medicine too. At the beginning of the sales in Great Britain, the lack of toxicity was used as a sale argument for the substitution of barbiturates for thalidomide, especially for pregnant women. In 1959, however, reports of newborn children with malformations emerged. Nonetheless, only in 1961, 2 pieces of research associated 34 cases of newborn children with malformations to the use of thalidomide by pregnant women in Germany. Similarly, in Australia, McBride presented 2 other studies that identified anomalies in various organs, called embryopathy [1,5].

Chemie Grünenthal withdrew the medicine from the market between the years of 1961 and 1963 in multiple countries after the researches were published. However, it is estimated that in this period around 10,000 children were born with congenital defects in the whole world; the number of abortions caused by the drug is unknown. The main identified defects were in the limbs, heart, ears, and eyes [2,5–7]. The identification of children with embryopathy resulting from the effects of thalidomide follows some essential patterns, which differentiates it from other congenital diseases. Specifically, this phenotype is defined as any bilateral upper and/or lower limb reduction defect of the preaxial and/or phocomelia types [3,8,9].

Controlled use of thalidomide has been an internationally important subject, since the late 1950s, when thousands of babies became victims of teratogenicity. Thalidomide toxicity triggered investments in the public sector and the pharmaceutical industry to promote greater safety and monitoring of drug production, marketing, and use [8,10,11]. Sixty years later, can we confirm that lessons have been learned and that the population is protected from this type of tragedy? Recent cases of pregnant women using thalidomide in Brazil indicate that the controlled use of thalidomide needs further attention.

Beginning in 1998, countries such as the United States of America started to follow patients who took thalidomide using computerized monitoring systems that were created by pharmaceutical companies. Although thalidomide has been approved for the treatment of erythema nodosum leprosum (ENL), the largest volume of distribution was registered for use in treating multiple myeloma (MM) [12,13]. Several European countries and Japan have also recorded MM as the primary use for thalidomide [14,15].

In Brazil, thalidomide is indicated for the treatment of ENL, aphthous ulcers in patients with HIV-AIDS, graft-versus-host disease, lupus erythematosus, MM, and myelodysplastic syndrome [16]. The treatment for patients affected by these diseases is overseen by the Unified Health System (*Sistema Único de Saúde* [SUS]) [17], which requires treatment to be organized following country regulations.

Leprosy is a public health issue that is hard to eliminate in Brazil, which is currently the second country in number of cases. The treatment prescribed in Brazil is the same one recommended by the World Health Organization (WHO), and it is offered for free by the SUS. Over the years, it is possible to observe that there has been, however slow, a tendency toward the diminishing in the incidence and prevalence of leprosy. The elimination of leprosy as a public health problem was achieved worldwide in 2000, based on the WHO Global Strategies introduction, implemented since the multidrug therapy introduction in the 1990s. Brazil, however, did not achieve the overall goal of less than one case per 10,000 inhabitants by 2015 [18–20].

The most recent WHO strategy (2016–2020) for eliminating leprosy is aimed at early detection and immediate treatment. This strategy was elaborate to achieve a long-term “leprosy-free world.” This refers to a situation wherein the community is free of morbidity, disabilities, and social consequences due to leprosy. Thus, the goal is to achieve zero disabilities in children diagnosed with leprosy, to have a Grade 2 Disability (G2D) rate of less than 1 case per million inhabitants, and to prevent any country from discriminating against a person with leprosy. For this reason, in countries where leprosy is endemic, the strengthening of government partnerships is crucial [19].

The interest for this study came about as a result of the limited number of analyses of the potential flaws in the control process of the use of thalidomide and of the factors that influence this challenging scenario in developing countries.

This article will discuss the control of thalidomide use in Brazil based on an analysis of the access regulation, the thalidomide consumption and distribution profile in the SUS, the epidemiological profile and population characteristics of leprosy, cases of embryopathy, and the occurrence, context, and consequences of thalidomide use during pregnancy.

## Methods

This study was designed to be a scoping review. This technique consists of “mapping” relevant studies in a specific area of interest and identifying the main concepts, theories, sources of evidence, and research gaps. The goal of a scoping review is to generate a broad understanding of the phenomenon being analyzed through systematic selection, collection, summarization of knowledge, and analytical interpretation. It is, therefore, an ideal technique to address questions that use various research methodologies [21]. Despite the broader design than that of a classic systematic review, our study of scoping review was submitted to a quality review according to Preferred Reporting Items for Systematic Reviews and Meta-Analyses (PRISMA) criteria [22].

All articles published in PubMed, Web of Science, and the Thesis & Dissertations Catalog of Coordination for the Improvement of Higher Education Personnel (CAPES) databases were searched on October 14, 2018. The Latin American and Caribbean Health Sciences Literature (LILACS) database was searched on February 16, 2019. All searches used the indexed descriptor “thalidomide” and retrieved 19,422 and 244 articles, respectively. The Scientific Electronic Library Online (SciELO) database was searched on March 30, 2019, using the indexed descriptors and their Portuguese version, “leprosy,” combined with “socioeconomic factors,” “socioeconomic,” “primary health care,” and “erythema nodosum.” A total of 42 articles were retrieved from this search.

The inclusion criteria defined in the selection were studies that worked with topics related to the following subthemes, in the Brazilian context: (1) organization of access to and use of thalidomide in the health system; (2) epidemiological and population characteristics of people with leprosy; and (3) occurrence of pregnancy and cases of embryopathy with the use of thalidomide. Clinical trials, pharmacokinetic studies, studies that dealt with international policies, duplicate or inaccessible studies, congress annals, and abstracts were excluded.

The following types of documents were also consulted: (1) Brazilian laws related to the regulation of thalidomide; (2) epidemiological data from the Notifiable Diseases Information System (*Sistema de Informação de Agravos de Notificação* [SINAN]); (3) epidemiological bulletin of the Ministry of Health (MoH) and WHO; and (4) protocols, manuals, and books published by the MoH and WHO that refer to public policies governing access to basic care, caring for leprosy patients, pharmaceutical assistance, and treatments using thalidomide. In addition, thalidomide consumption data were collected from the MoH, as allowed under the Access to Information Law (No. 12.527/2011).

**Fig 1:**
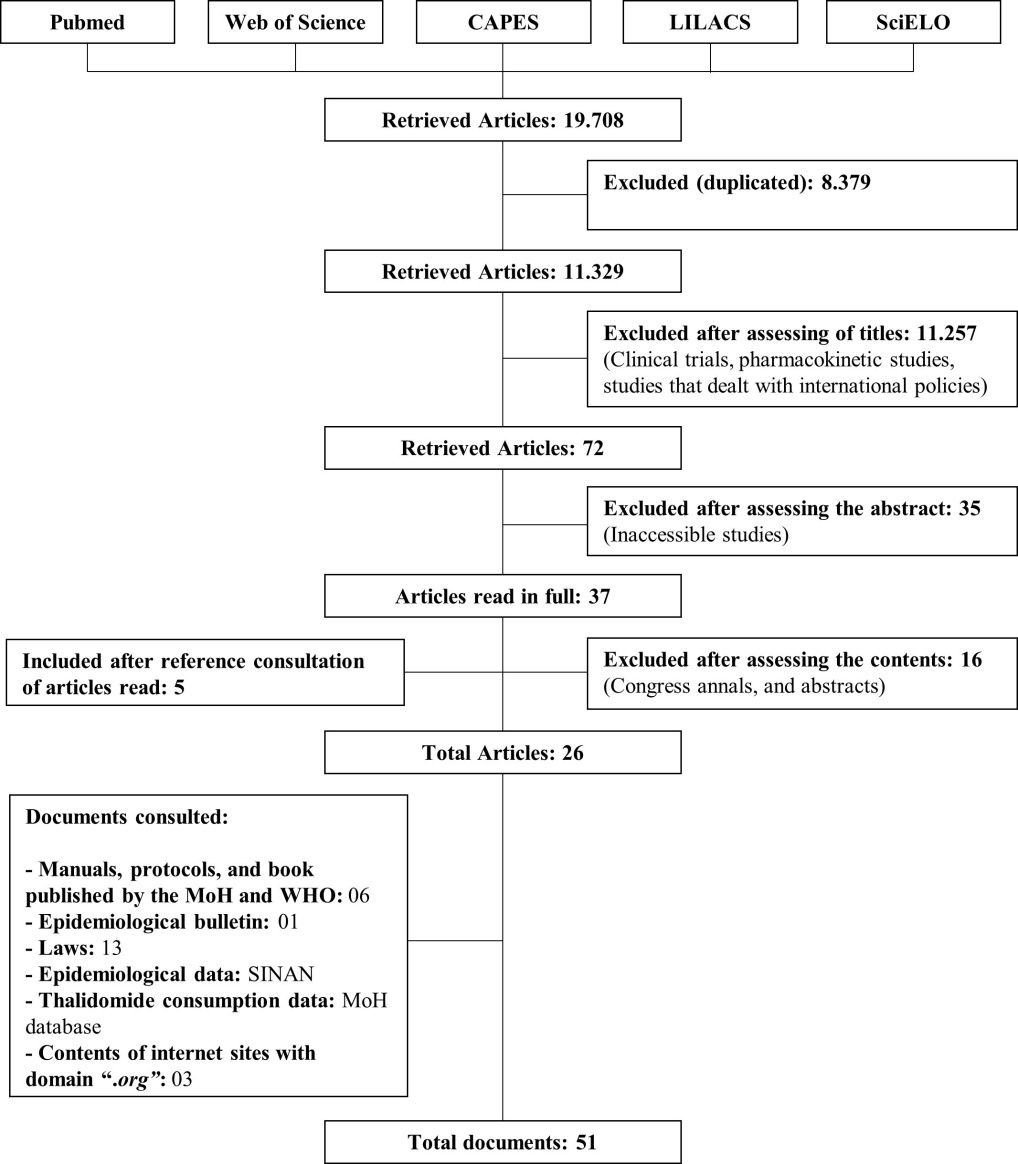
Flowchart for the handling of studies during the review process. CAPES, Coordination for the Improvement of Higher Education Personnel; LILACS, Latin American and Caribbean Health Sciences Literature; MoH, Ministry of Health; SciELO, Scientific Electronic Library Online; SINAN, Notifiable Diseases Information System (*Sistema de Informação de Agravos de Notificação*); WHO, World Health Organization.

In total, 51 documents met the inclusion criteria (Fig 1). This included 23 articles published in scientific journals [1,8,9,11,23–41], 3 postgraduate studies sensu stricto [3,4,10], 6 protocols, manuals, or books published by the MoH and WHO [18–20,42–44], 1 epidemiological bulletin [45], 13 laws [17,46–57], data from 2 databases of the MoH [58,59], and content from 3 internet sites with the domain “.*org*” [60–62]. Secondary data are subject to underreporting and failures af recording systems.

## Results

### Organization of access to thalidomide in the health system

In 1958, thalidomide entered the Brazilian market for the treatment of several clinical conditions. It remained on the market until 1962 when its marketing was suspended because of teratogenicity cases [10]. In 1965, the use of thalidomide in Brazil was approved for the treatment of ENL [23]. However, regulations that outlined the control of thalidomide use in the country were published decades later. They regulated the registration, production, manufacture, marketing, exposure to sale, prescription, and dispensing of thalidomide-based drugs (Decree No. 354/1997), and substances and medicinal products (including thalidomide) that are subject to special control (Decree No. 344/1998) [46,47].

In 2003, a law that requires mandatory control and supervision of thalidomide use in Brazil (Law no. 10.651) was promulgated and determined that the thalidomide use is subject to special control and sanitary supervision rules published by the National Health Surveillance Agency (*Agência Nacional de Vigilância Sanitária* [ANVISA]). This law also enabled the reformulation of the decree that regulated the thalidomide in 1997 (Decree No. 354/1997) to the current Resolution of the Board (RDC) of the ANVISA (RDC No. 11, dated 03/22/2011). The publication of this legislation reflected the articulation of civil society and was based on the regulatory processes of other countries. In the end, this resulted in shared responsibility between public laboratories, health managers including MoH and ANVISA, health professionals, and patients [17,48].

Following the implementation of these regulations, the organization of access to thalidomide in the SUS is as follows: (1) the drug is produced exclusively by a public laboratory and purchased in a centralized manner by the MoH, to be made accessible solely through those programs available in the SUS; (2) the MoH began distributing thalidomide to the State Health Secretariats in accordance to the consumption in the public healthcare system; (3) the state’s Pharmaceutical Services became responsible for distributing thalidomide to public facilities that possessed valid accreditation, as well as maintaining the patient registry and forwarding their data to the MoH national bank. The RDC allows records to be manual or computerized; (4) thalidomide became dispensed only by a pharmacist, who is responsible for all activities related to the pharmaceutical service within the accredited public health centers; (5) the patient is required to present a “thalidomide prescription notification” and a “clarification and responsibility term” at the time of drug dispensing to have access to thalidomide. This form must be filled and signed by both the registered physician and the patient. Women of childbearing potential must demonstrate the use of effective contraception and pregnancy tests; (6) the amounts dispensed from thalidomide may not exceed 30 days of treatment, and the “thalidomide prescription notification” is valid for 20 days; (7) the local health authority is required to register prescribing doctors and to accredit public health centers. This accreditation is based on requirements such as having the necessary documents for operation, up-to-date sanitary licenses, facilities compliant with current legislation, responsible pharmacists and professionals trained on control activities, as well as conducting an annual evaluation. The local health authority is also responsible for acts related to the use, production, manufacture, packaging, repackaging, marketing, distribution, transport, storage, prescription, dispensing, disposal, or any other activity related to thalidomide and to enforce the determinations of federal legislation; (8) the ANVISA does postmarket surveillance of thalidomide, and it is mandatory that any adverse event related to its use must be notified to the federal agency; and (9) the Brazilian government should promote permanent education campaigns regarding the risks of thalidomide [17].

ANVISA initiated a national training program in 2011 to train professionals to follow RDC No. 11/2011 [60], which continued until 2014 and reached all the states of Brazil [61]. After 2014, ANVISA and MoH performed the training activities together, promoting the 1^st^ National Meeting on the Control of Thalidomide in 2015; the publication of educational materials for health professionals and patients; the implementation of technical visits to various states to assist in organizing services in 2016; and, between 2014 and 2016, the mapping of all public thalidomide dispensing health centers in the country. This last activity was conducted with local health authorities, in order to understand how access was organized [62].

**Fig 2:**
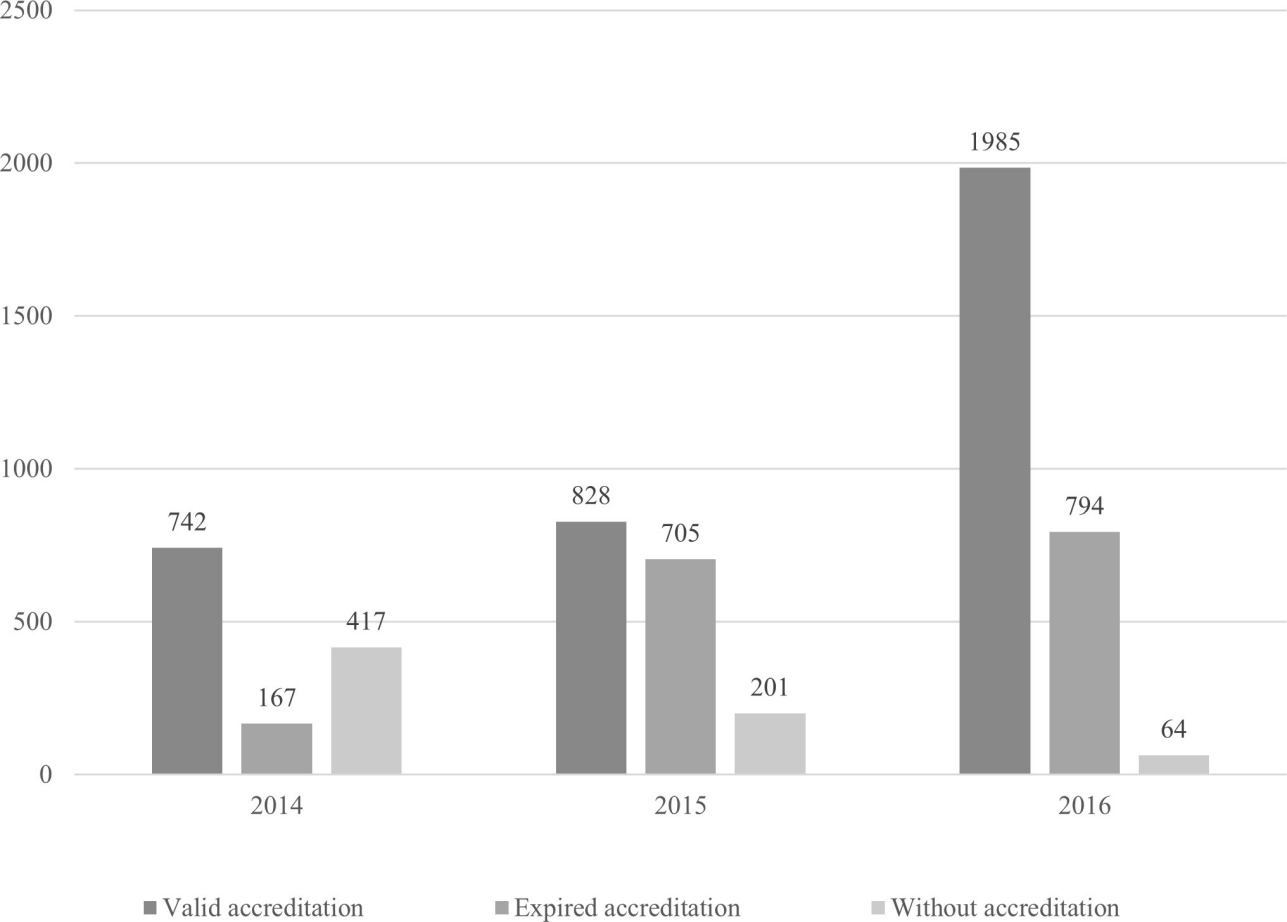
Number of public health centers dispensing thalidomide in Brazil between 2014 and 2016 [58].

In reference to the mapping of public health centers in the states, the reported data covered both primary care and specialized care centers. These health centers included those with valid accreditation, expired accreditation, after the expiry of the validity of 1 year, and those that did not have accreditation but dispensed thalidomide (Fig 2).

In 2014, this study identified 1,326 health centers distributed across 22 states of Brazil. The following year, this number increased to 1,734 health centers in 26 states, and in 2016, it increased even further to 2,843 health centers in 22 states. Not all states responded, which was partially justified by the lack of records from the local health authorities [58].

An increase in the number of public health centers with valid accreditation was observed, particularly in 2016. There was also a considerable decrease in the number of health centers that dispensed thalidomide without permission from the local health authority, even though there were still some registers of such.

### Profile of consumption and distribution of thalidomide in Brazil

The benefits of thalidomide in different clinical conditions has been sought since the 1960s [1,24]. In 1965, after the discovery of the efficacy of thalidomide in the treatment of ENL [23], Brazil allocated its use exclusively for leprosy [1]. In the 1990s, thalidomide use was expanded to the treatment of systemic lupus erythematosus, aphthous ulcers in patients with HIV/AIDS, and graft-versus-host disease. It was also approved for treating MM in 2000 and for myelodysplastic syndrome in 2015 [3,10].

Thalidomide is listed in Annex II of the National List of Essential Medicines, which is part of the Strategic Component of Pharmaceutical Services (*Componente Estratégico da Assistência Farmacêutica* [CESAF]). The CESAF is intended to guarantee equitable access to medicines and supplies for prevention, diagnosis, treatment, and control of diseases, ailments, or conditions with endemic profile, with epidemiological importance, socioeconomic impact or affecting vulnerable populations, covered in strategic programs of health of the SUS [42].

Recommendations on the use of thalidomide were published by the MoH, as well as the Clinical Protocol and Therapeutic Guidelines (*Protocolo Clínico e Diretrizes Terapêuticas* [PCDT]) or the technical manuals; and recommendations on issues of control and sanitary supervision were published by ANVISA. In 2015, the clinical indications were updated by ANVISA (RDC No. 50, on 11/11/2015) (Table 1).

**Table 1:** Clinical indications approved for use of thalidomide in Brazil.

Clinical Indication	Technical Information	Description of the Manual or PCDT	Regulations in Force
Erythema nodosum leprosum	Thalidomide is the first-line drug used in treatment. Alternative therapies: prednisone and pentoxifylline.	Technical-operational manual provides guidelines for surveillance, attention, and elimination of leprosy as a public health problem [49].	Consolidation Decree No. 2, 09/28/2017.
Aphthous ulcers in patients with HIV/AIDS		Pharmaceutical Assistance protocol in patients with HIV/AIDS [50].	Consolidation Decree No. 6, 09/28/2017.
Systemic lupus erythematosus	Thalidomide is not a first-line drug for treatment. Alternative therapies include chloroquine, hydroxychloroquine, methylprednisolone, cyclosporine, azathioprine, cyclophosphamide, methotrexate, and danazol.	Approves the Clinical Protocol and Therapeutic Guidelines of Systemic Lupus Erythematosus [51].	Decree of the Health Attention Secretariat/MoH No. 100, 02/07/2013.
Graft-versus-host disease	Used in patients who are refractory to first-line treatment with corticosteroids.	Updates the protocols for use of thalidomide in the treatment of disease of graft-versus-host and multiple myeloma [52].	Decree of the Health Care Secretariat/MoH No. 298, 03/21/2013.
Multiple myeloma	Used as a first-line treatment or in refractory patients.		
Myelodysplastic syndrome	Used in patients who are refractory to erythropoietin.	Approves The Clinical Protocol and Therapeutic Guidelines of Myelodysplastic Syndrome [53].	Decree of the Health Attention Secretariat/MoH No. 493, 11/06/2015.

MoH, Ministry of Health; PCDT, Clinical Protocol and Therapeutic Guidelines (*Protocolo Clínico e Diretrizes Terapêuticas*).

In this context, both federal agencies (MoH and ANVISA) follow the prerogatives of the current laws regarding recommendations for care of a patient in the public network and structuring of services (Law No. 10.651/2003, RDC No. 11/2011), in particular, the law that regulates assistance therapy and the incorporation of health technology within the SUS (Law No. 12.401 of 04/28/2011) and the law that controls the organization of the SUS, the planning of health, healthcare, and interfederative articulation, taking additional action to regulate health (Decree No. 7.508 of 06/28/2011, which regulates the Law No. 8.080) [17,48,54,55].

According to current legislation (Article 15 of the RDC No. 11/2011), it is the responsibility of the MoH to create and maintain the “National Registry of Patient of Thalidomide-Based Drugs,” which should contain detailed information about the patient, the prescriber, and the dispenser, as well as the treatment. However, the MoH does not yet have this database. Specifically, there is no concrete information on the patient registry in the public network or information on the control of thalidomide dispensation [17,58].

**Fig 3:**
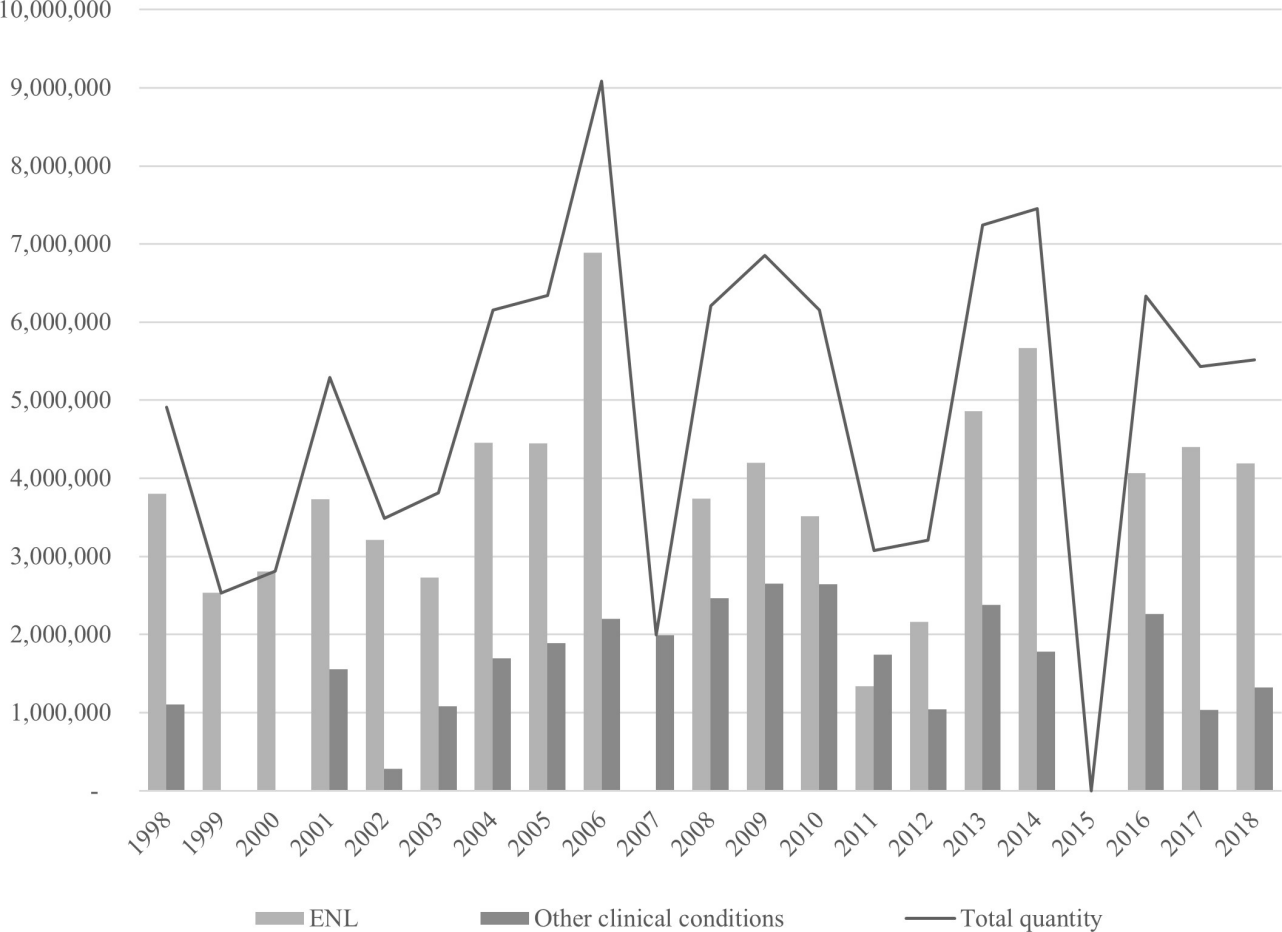
Number of 100 mg thalidomide tablets acquired by the Brazilian Ministry of Health for the treatment of ENL and other clinical conditions between 1998 and 2018. Other clinical conditions (lupus, multiple myeloma, aphthous ulcers in patients with HIV-AIDS, graft-versus-host disease, and myelodysplastic syndrome). [58] ENL, erythema nodosum leprosum.

Data on consumption of thalidomide in the public network are based on drug purchase records [58]. Between 1998 and 2018, the MoH acquired 103,889,280 tablets from the Ezequiel Dias Foundation (*Fundação Ezequiel Dias* [FUNED]), the only public laboratory producing the drug in Brazil. The tablet unit price increased from BRL$0.16 in 1998 to BRL$1.41 in 2018. Seventy percent of these tablets were then allocated to the leprosy program (Fig 3).

Between 1998 and 2002, when the laws that regulated the control of thalidomide and substances subject to special control were in force (Decree No. 354/1997 and Decree No. 344/1998), 19,031,520 thalidomide tablets were purchased, an average of 3.8 million tablets were consumed annually. Of the total tablets purchased, approximately 85% were intended for the treatment of patients with ENL (16,092,960 tablets).

From 2003 to 2010, the number of tablets purchased increased to 46,591,680, which represented approximately 5.8 million tablets/year, about 53% more than in the previous period. Although ENL was the most common clinical indication for thalidomide use (64%), there was a significant (253.5%) increase in average annual consumption for other clinical conditions (lupus, MM, aphthous ulcers in patients with HIV-AIDS, and graft-versus-host disease). These years were marked by the promulgation of a law which made controlled use of thalidomide mandatory (Law No. 10.651/2003).

From 2011 to 2018, there was a decrease in the number of tablets purchased when compared with the previous 8 years, even though thalidomide was approved for the treatment of myelodysplastic syndrome in 2015. The total number of tablets purchased in this period was 38,266,080, an annual average of 4.7 million tablets (approximately 18% less than the previous period). Again, it can be observed that the leprosy program still showed the highest portion of consumption, accounting for approximately 70% of the total acquired tablets (26,691,840 tablets).

**Fig 4:**
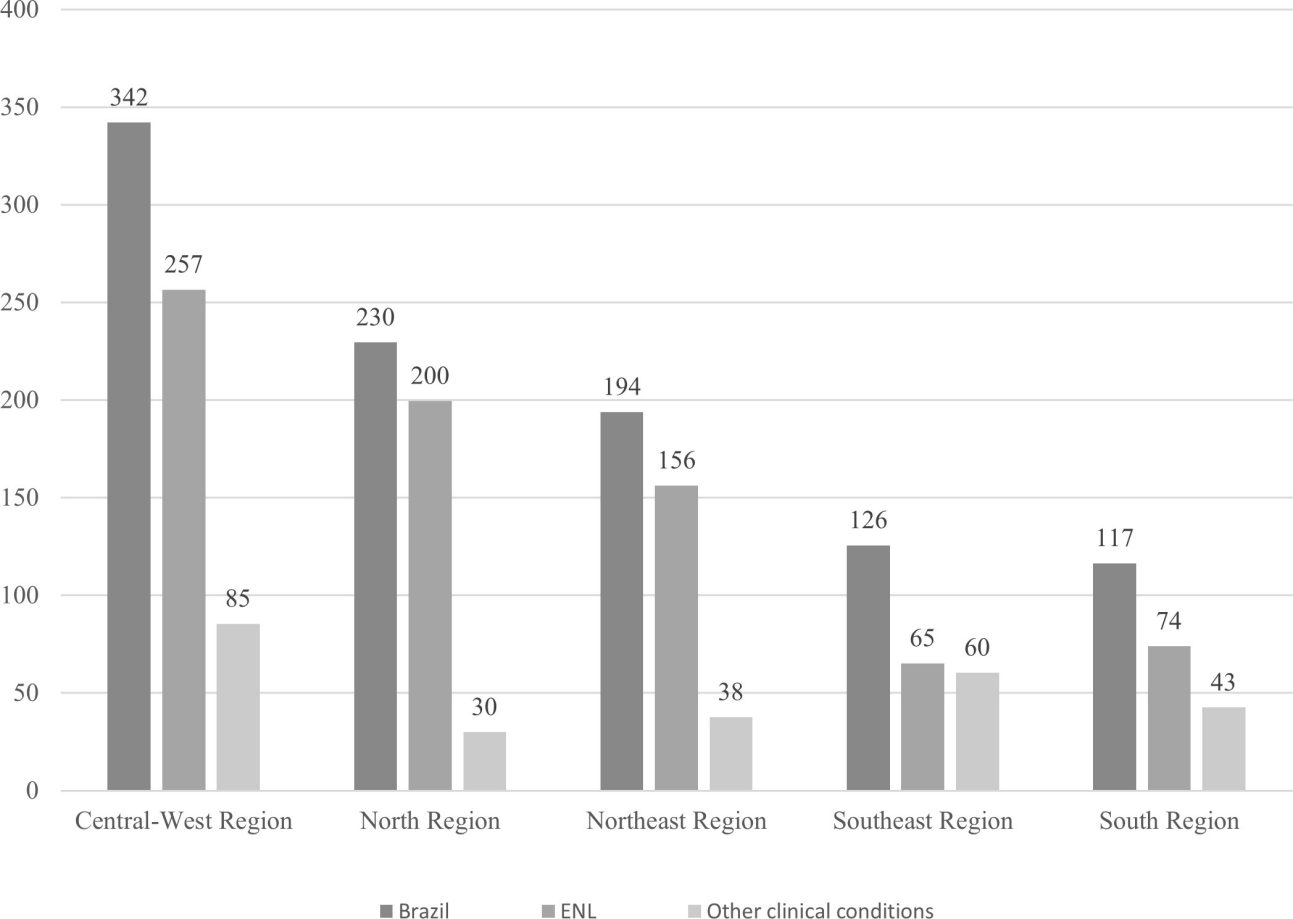
Average rate of distribution of 100 mg thalidomide tablet/10 thousand inhabitants among the Brazilian regions, from 2011 to 2018. Average number of 100 mg thalidomide tablets in the state between 2011 and 2018/average total population in the state between 2011 and 2018 per 10,000 inhabitants. Other clinical conditions (lupus, multiple myeloma, aphthous ulcers in patients with HIV-AIDS, graft-versus-host disease, and myelodysplastic syndrome) [58,63]. ENL, erythema nodosum leprosum.

According to the MoH, from 2011 to 2018, 27,230,440 thalidomide tablets were distributed to states, of which 19,004,080 tablets went to the care of patients with ENL. The average rate of distribution of thalidomide among Brazilian regions is shown in Fig 4. The Central-West, the North, and the Northeast Regions showed the highest rates. The average rate of thalidomide distribution for ENL was higher than for other clinical conditions in all regions.

### Epidemiological profile of leprosy and population characteristics

The relationship between the consumption of thalidomide and the treatment of ENL indicates a need to evaluate the epidemiological and demographic profile of leprosy in Brazil.

Leprosy is a chronic infectious disease in which diagnosed cases are classified according to the clinical presentation (indeterminate, tuberculoid, dimorphic, or lepromatous) and the number of cutaneous lesions (multibacillary or paucibacillary) [18,25].

According to WHO, in 2017, Brazil, India, and Indonesia collectively contributed 80.2% of the global burden of new cases of leprosy. Overall, 210,671 new cases were reported by 150 countries that year, with a general detection rate of 2.77 cases per 100,000 inhabitants. Of these cases, 26,875 were Brazilian, with a detection rate of 12.94 cases per 100,000 inhabitants. There were 1,718 new cases recorded in patients younger than 15 years old, with a detection rate of 3.72 cases per 100,000 inhabitants. According to the detection rate references, both rates are considered high [45,59].

In addition, in 2017, the rate of G2D was 8.30 cases per 1,000,000 inhabitants, indicating a late diagnosis in the population. Although approximately 80% of these cases were cured, the registered prevalence was 1.35 cases per 10,000 inhabitants [45,59].

**Fig 5:**
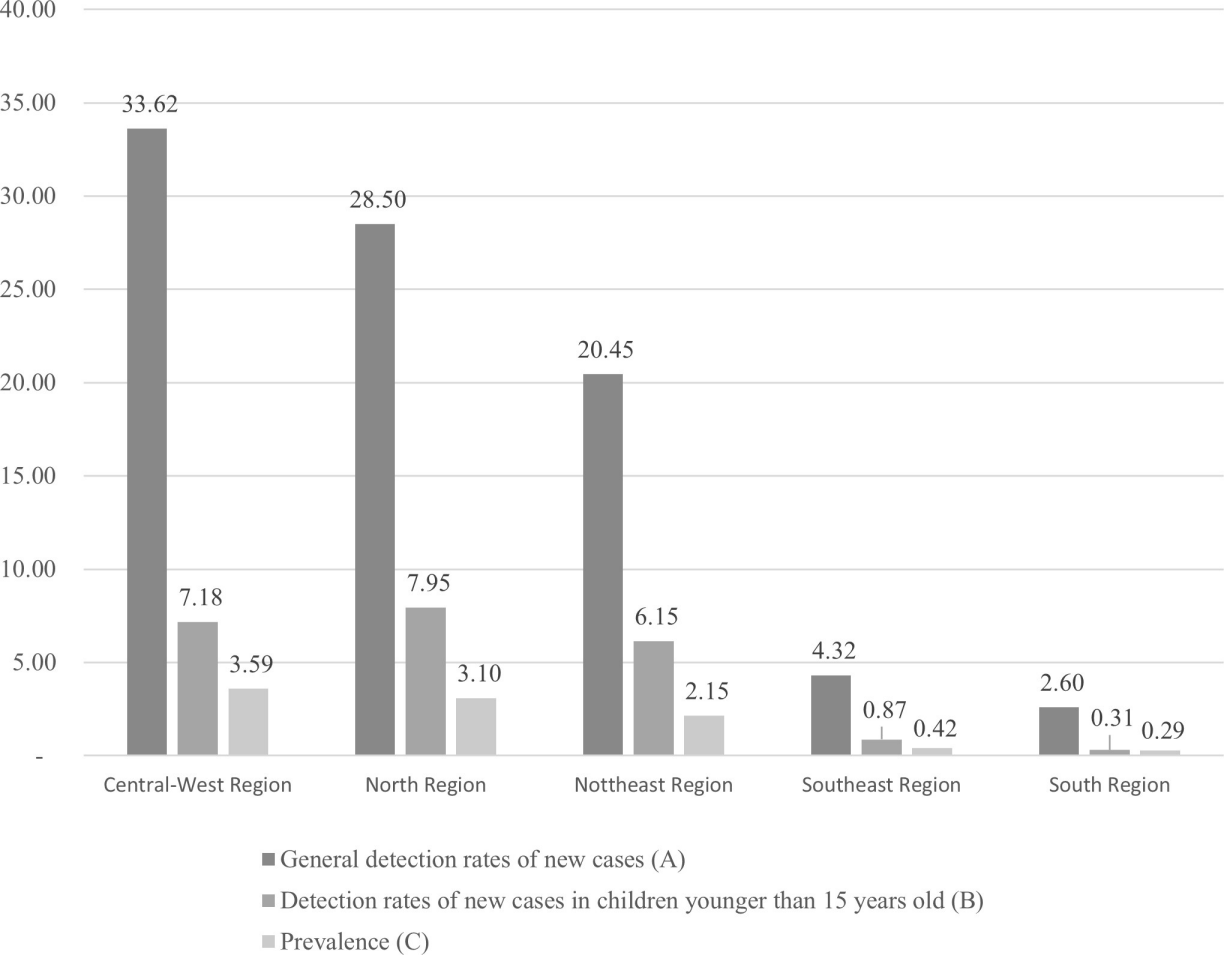
General detection rates of new cases, detection rates of new cases in children younger than 15 years old and prevalence, among the Brazilian regions, in 2017. (A) Number of leprosy new cases in the region in 2017/total population in the region in 2017 per 100,000 inhabitants. (B) Number of leprosy new cases in children younger than 15 years old in the region in 2017/total population of the children younger than 15 years old in the region in 2017 per 100,000 inhabitants. (C) Number of leprosy cases in treatment in the region in 2017/total population in the region in 2017 per 10,000 inhabitants. [59].

In Brazil, all states have cases of leprosy. However, the Central-West, the North, and the Northeast regions are particularly notable because they have the highest risk. Accordingly, these regions have high leprosy prevalence and high rates of general detection of new cases in children younger than 15 years old, as shown in Fig 5 [59].

Care for the people affected by leprosy occurs within the scope of the SUS, and basic treatment is provided to adults and children in accordance with the operational classification. Because the presence of bacilli, some people may experience alterations in the immune system and develop reactional episodes, which can appear before, during, or after the basic treatment. These reactions can be type 1 or type 2, which is characterized by reddish and scaly lesions or the presence of painful subcutaneous nodules, respectively [18,20].

ENL, a frequent manifestation of type 2 reactions, occurs only in multibacillary-infected patients who have lepromatous type and dimorphic leprosy. It is estimated that 37% of the cases with multibacillary leprosy present as ENL [25]. In 2017, for example, this represented 8,993 people across the country, of a total of 24,306 cases diagnosed with multibacillary leprosy [59].

### Embryopathy cases due to thalidomide use during pregnancy

One important finding from the studies was that the type of anomaly was directly related to the day thalidomide was taken during pregnancy, although all doses caused defects [8].

In Brazil, approximately 300 cases of malformations due to thalidomide use were identified until 1965 [1]. Gollop and colleagues (1987) reported the case of a woman who had leprosy and needed to take thalidomide, which she used until the 35th day of gestation [26]. After severe phocomelia was identified, the couple decided to interrupt the pregnancy [26,27]. Between 1969 and 1995, 34 cases of malformation due to thalidomide were reported in South America, of which 33 cases were Brazilian. In 10 cases reported to the Latin-American Collaborative Study of Congenital Malformations (*Estudo Colaborativo Latino-Americano de Malformação Congênita* [ECLAMC]), all women had leprosy and took thalidomide during pregnancy [9]. In 2005 and 2006, 3 cases were registered in the Porto Alegre Teratogenic Agents Information Service (*Serviço de Informações sobre Agentes Teratogênicos* [SIAT]), occurring in the states of Rondônia, Rio Grande do Sul, and Maranhão. In 2 cases, the women took thalidomide prescribed to another person [28].

The resurgence of embryopathy led a group of researchers to conduct proactive monitoring between 2000 and 2008 in selected hospitals, to identify birth defects consistent with the thalidomide embryopathy phenotype (TEP). The method was considered sensible and feasible because the clinical research identified 2 cases of newborns with TEP in the same hospital in the Northeast of Brazil. Although maternal intake of thalidomide was not demonstrated, researchers associated the increase of TEP with the availability of thalidomide and the frequency of leprosy cases [29].

This same study was replicated for all births in the country between 2005 and 2010, using data on the prevalence of leprosy and the distribution of thalidomide in the same period. There was a direct correlation between the amount of thalidomide distributed and the number of children born with TEP, suggesting the need for emergency education on the safe use of the drug [30].

In 2013, 2 more cases were identified. In both cases, exposure to thalidomide happened as a result of ENL treatment [3,31]. Table 2 provides an overview of the cases registered in Brazil.

**Table 2:** Reported cases of embryopathy registered in Brazil, from 1965 to 2010.

Year	State/Region	Cases reported	References
Up to 1965	Several states	Approximately 300 cases throughout Brazil. The drug was withdrawn from the market in 1962.	[1]
1987	São Paulo (Southeast Region)	One case identified by the service of Human Genetics of the Maternity Hospital Association of São Paulo. The woman had leprosy and took thalidomide until the 35th day of gestation. The pregnancy was interrupted.	[26]
1988	São Paulo (Southeast Region)	One case identified by the MORHAN.	[43]
1992		21 cases identified by MORHAN and a British television network (Yorkshire Television), and 26 cases identified after the release of the documentary “*Thalidomide*: *the drug that came back*.”	[27,32,43]
1993		65 cases identified by MORHAN and by the ABPST.	[43]
1996	Several states (North, Northeast and South Regions)	33 cases identified in the states of São Paulo, Rio de Janeiro, Minas Gerais, Pernambuco, Bahia, Amazonas, Pará, Paraná, and Rio Grande do Sul. One child was born in 1969, 6 in the 1970s, 20 in the 1980s, and 6 in the 1990s. Of the 10 cases reported to the ECLAMC, all women had leprosy and took thalidomide during pregnancy.	[9]
1998	Maranhão (Northeast Region)	Birth of a male child with malformations in the upper and lower limbs. The woman was treated for leprosy in 1995 and presented with ENL. However, she did not remember the exact dose and for how long she used thalidomide during pregnancy.	[3,31]
2005	Rondônia (North Region)	Birth of a male child with malformations in the upper and lower limbs. The husband took thalidomide for ENL. The woman took thalidomide without being aware of her pregnancy. The case was reported to the DEVEP of the MoH. Technical visits were carried out to investigate the case.	[28,43]
2006	Maranhão (Northeast Region)	Birth of a female child with malformations in the upper and lower limbs. She died due to severe congenital heart disease. The woman had taken thalidomide for the treatment of ENL since 2003. The case was reported to the DEVEP of the MoH. Technical visits were carried out to investigate the case.	[28,43]
2006	Rio Grande do Sul (South Region)	Birth of male twins with malformations. The first one died due to defects in the urinary tract and pulmonary hypoplasia. The second had bilateral symmetrical defects in the upper limbs. The woman was 17 years old, did not desire this pregnancy, and took thalidomide prescribed to her mother for MM treatment. The case was notified to DEVEP of the MoH. Technical visits were carried out to investigate the case.	[28,43]
2007	Northeast Region	Two cases identified with TEP, although ingestion of thalidomide by the women was not shown.	[29]
2010	Maranhão (Northeast Region)	Birth of a female child with malformations in the upper and lower limbs. The woman took thalidomide to treat ENL in the previous year. As she did not use contraceptives, thalidomide was suspended. However, she had ingested several tablets at home before knowing that she was pregnant.	[3,31]

ABPST, Brazilian Association of Thalidomide Syndrome Carriers (*Associação Brasileira de Portadores de Síndrome de Talidomida*); DEVEP, Department of Epidemiological Surveillance (*Departamento de Vigilância Epidemiológica*); ECLAMC, American Collaborative Study of Congenital Malformations (*Estudo Colaborativo Latino-Americano de Malformação Congênita*); ENL, erythema nodosum leprosum; MM, multiple myeloma; MoH, Ministry of Health; MORHAN, Movement of Reintegration of People Affected by Leprosy (*Movimento de Reintegração das Pessoas Atingidas pela Hanseníase*); TEP, thalidomide embryopathy phenotype.

In compliance with Brazilian laws (Law No. 7.070 of 12/20/1982 and Law No. 12.190 of 01/13/2010), the federal government should grant a lifetime special pension and indemnity to people with physical disabilities that occurred as a consequence of thalidomide use [56,57]. However, the literature has given little attention to cases of congenital malformation and their correlation with the use of thalidomide when compared with the benefits provided by the federal government. Santos (2018) pointed out that 1,073 victims of thalidomide were identified until 2010. These individuals were compensated by the federal government and were spread over all regions of the country: 27 in the North, 143 in the Northeast, 150 in the Central-West, 533 in the Southeast, and 220 in the South [4].

Between 2015 and 2018, 5 new cases of use of thalidomide during pregnancies were reported to the MoH and ANVISA. These cases are currently under investigation and occurred in the state of Piauí in 2015, the states of Maranhão and Pernambuco in 2017, and the states of Goiás and Minas Gerais in 2018 [58].

**Fig 6:**
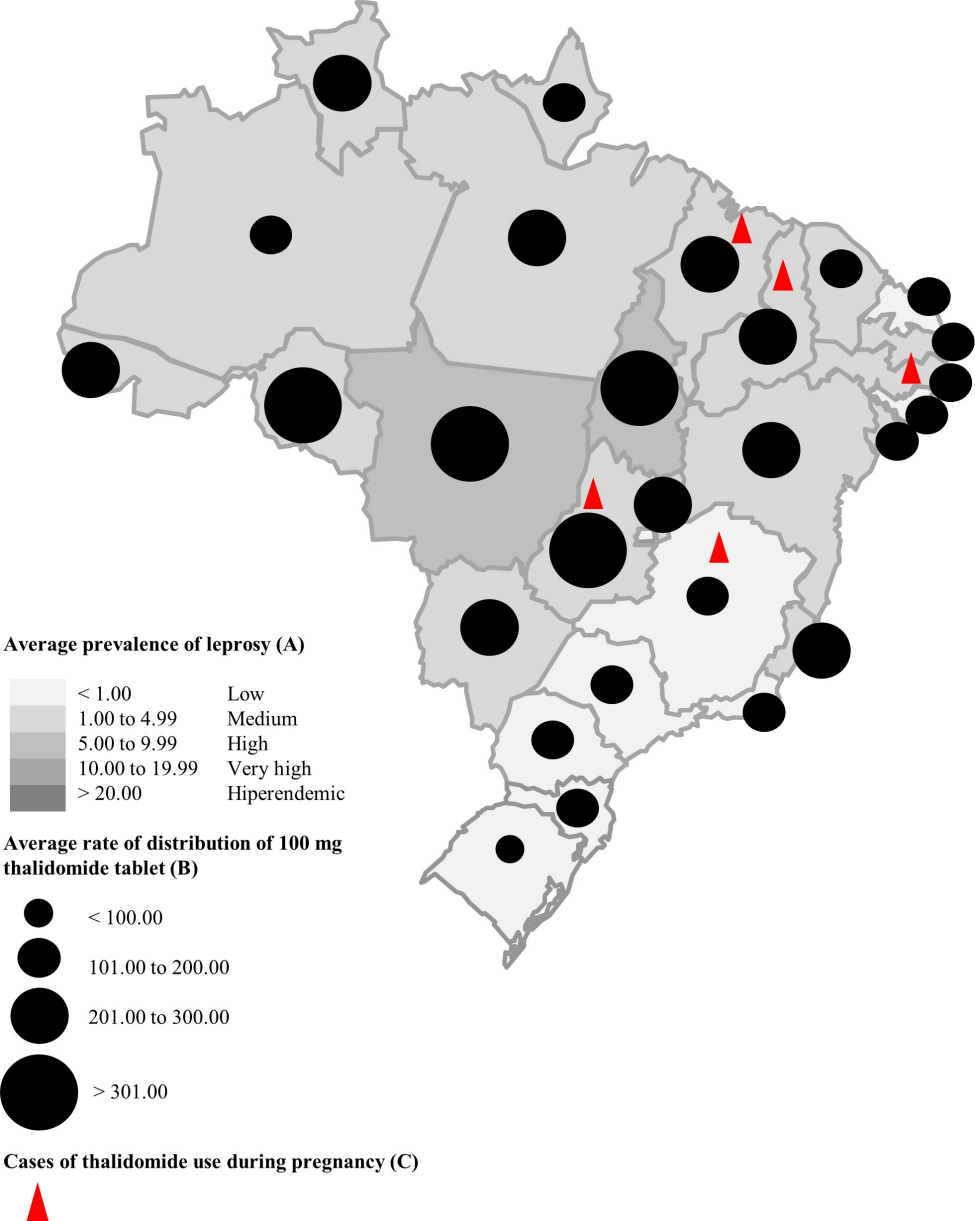
Average prevalence of leprosy, average rate of distribution of 100 mg thalidomide tablet, and reported cases of thalidomide use during pregnancy in Brazil between 2011 and 2018. (A) Average number of leprosy cases in the state between 2011 and 2018/average total population in the state between 2011 and 2018 per 10,000 inhabitants. (B) Average number of 100 mg thalidomide tablets in the state between 2011 and 2018/average total population in the state between 2011 and 2018 per 10,000 inhabitants [58,59,63].

In other words, since the publication of the law that regulates the control of thalidomide use (RDC No. 11/2011), 5 cases of thalidomide use during pregnancy were identified in Brazil, and of these, 4 have occurred in 2 endemic regions for leprosy and with high rates of consumption of thalidomide—the Northeast region and the Central-West region (Fig 6).

## Discussion

The analysis of documents and literature regarding the use of thalidomide in Brazil in recent years indicates that there are 2 extremely worrying and important issues emerging: one is the control of thalidomide distribution and use; the other is the control of leprosy as a triggering factor for use of thalidomide.

The present study reveals that Brazil took nearly half a century to regulate the use of thalidomide. The analysis of the profile of use for this drug demonstrates that although thalidomide is indicated for the treatment of several clinical conditions in Brazil, only leprosy is considered a neglected disease. The guidelines regarding the proper care of leprosy patients are contained within a technical-operational manual published by the MoH. Under these guidelines, it is up to the prescriber to choose the dose of thalidomide used in the treatment of ENL (100 to 400 mg). In this context, leprosy is the only clinical condition that does not have a PCDT with well-defined criteria for the diagnosis, monitoring, and discontinuation of thalidomide treatment, that are available to managers, health professionals, and patients. This perception corroborates the findings of Santana and colleagues (2018), who evaluated the methodological quality of documents published by the MoH and pointed out that all poverty-related disease guidelines were considered “not recommended” because “they did not meet the minimum criteria of the scientific method description, failing to provide whether or not the document was drawn up from an evidence-based health perspective,” or in other words, according to the PCDT legitimized by the publication of Brazilian laws (Law No. 12.401/2011 and Decree No. 7.508/2011) [64].

Although there is no PCDT for leprosy, the distribution rate and amount of thalidomide acquired by MoH indicate that there is high consumption of thalidomide in the SUS arising mainly from ENL cases. Despite the extensive use, WHO does not recommend thalidomide for ENL [23,65]. This high consumption, however, can not be confirmed through attendance data because the Thalidomide Users National Database is not yet operational. This suggests that the central domain of the MoH is to purchase and distribute the drug to the states. Consequently, one can infer that there is no way to trace the patient, the profiles of drug use, clinical and demographic conditions, or the dispensing process. It becomes impossible for managers to monitor and evaluate the services offered and make decisions about their effective functioning, as well as evaluate the entire system [66,67]. A factor that contributes indirectly to the absence of such database is that the public health centers may not register thalidomide patients at all because the law allows a manual registration of these patients’ data (RDC No. 11/2011) [17].

Regarding the accreditation of public health centers, the data showed that there were a larger number of health facilities regularized with the local health authority. Although there was a decrease in the number of health centers without accreditation at the end of the third year of mapping, there were still irregularities. This situation is not permitted by law and was reported by the local health authorities themselves. The lack of compliance in accreditation is worrying, because it demonstrates poor performance by the local health authorities in accrediting, supervising, and authorizing public health centers to dispense thalidomide. Moreover, as there are flaws in the accreditation process, some issues regarding facilities’ conditions may be present, which could endanger patients’ safety. This situation may be worse considering the precariousness of the health service's ability to manage the organization, operation, and maintenance of pharmaceutical services [68]. This process is essential for the development of public policies to control thalidomide use. This results in the provision of low-quality services and often without the participation of a pharmacist.

The 2015 National Survey on Access, Use, and Promotion of Rational Use of Medicines in Brazil (*Pesquisa Nacional sobre Acesso*, *Utilização e Promoção do Uso Racional de Medicamentos* [PNAUM]) evaluated the access and rational use of medicines by the Brazilian population and its effectiveness in primary healthcare in the SUS. Data from this survey illustrate the current situation of the system, in which only 40% of the primary healthcare centers evaluated in Brazil were reported as having a sanitary license to operate, and only 43% of the health centers had a responsible pharmacist. This study also noted that 60% of pharmacies in the primary healthcare centers did not have an environment conducive to care and lacked fundamental tools for implementing pharmaceutical activities (53% were without a computer, 44% were without internet access, and 58% of records were kept manually). The North and the Northeast regions were the least economically developed, but they received more resources from the National Program of Qualification of Pharmaceutical Services in the SUS (*Programa Nacional de Qualificação da Assistência Farmacêutica no Sistema Único de Saúde* [Qualifar-SUS]) for improvement, implementation, and systemic integration of pharmaceutical services activities [68–70].

Another aspect that called our attention is the use of thalidomide without a prescription. Of the cases reported in the literature of women who became pregnant during the use of thalidomide, 3 used a drug prescribed to another person. This attitude can be explained by the sociocultural context of the individual based on their knowledge and experiences in the health-disease-care process, which influences the practice of self-medication. Self-medication is one of the most widespread practices in society and therefore merits an understanding by health professionals of the relationship between culture, health-disease-care processes, and the use of medications [71]. Arrais and colleagues (2016) performed a study of the prevalence of self-medication in Brazil between 2013 and 2014 and reported that the Brazilian population follows this practice. This study found that self-medication was influenced by females and by the presence of diseases or chronic conditions and was more prevalent in the Northeast, the Central-West, and the North regions of the country [72].

Regarding the control of leprosy, the epidemiological data show high general detection rates, prevalence, and G2D in Brazil, which is the only country in the Americas where the disease is endemic [45]. These results can be attributed to the efforts of the MoH to detect the disease. One example of this was the creation of the project (2017–2019) “Innovative approaches to intensify efforts for a Brazil free of leprosy,” in partnership with the Pan American Health Organization (PAHO/WHO). This project aimed to train primary healthcare professionals to be able to diagnose and treat leprosy cases, with a focus on 20 priority municipalities in more endemic regions to new cases diagnosed in children younger than 15 years old (Maranhão, Mato Grosso, Pará, Pernambuco, Piauí, and Tocantins) [73]. However, the diversity of regional contexts and even between populations of the same region maintains the burden of the disease a hard one to overcome. Precarious health, high household occupancy rates, low economic and educational levels, and lack of food and sanitation directly influence the risk of becoming ill. Therefore, areas with these characteristics have more cases of leprosy, which is reflected in the general detection rate of new cases [18,33–37]. The indicator of the rate of G2D, for example, suggests not only the ability of a team to detect leprosy early but also the level of awareness of signs of leprosy and access to services. Because the reduction of this rate was not achieved, it can be inferred that there are flaws in the process of actively searching for cases, the training of health professionals, and the access to diagnosis and treatment. Late diagnosis suggests a hidden prevalence of disease and continued transmission, as well as the presence of uncured cases in the network [18,19,38].

Primary healthcare is the gateway for people affected by leprosy. For this reason, individual, family, and collective health actions must be guaranteed under the National Primary Health Care Policy [44]. With family health as its main strategy, primary healthcare should apply greater efforts in the healthcare networks to increase service levels, increase resolution, and coordinate actions so that the whole system works efficiently. This process requires the involvement of managers, health teams, and professionals who are committed to producing results that impact the main indicators of health and quality of life in the population [74]. One of the major challenges for Brazil is to have quality services and adequate infrastructure in line with public policies. Some studies that have evaluated control of leprosy in primary healthcare have identified the fact that social determinants of the disease and the status of endemicity are not the only factors that influence the risk of people getting sick. Organization of services is directly correlated with the process of healing and with the improvement of quality of life. In these studies, the factors that influenced risk of illness included a lack of adequate structure, scarce resources, absence of reference service, low autonomy and management resolution, fragility in the information system, precariousness of epidemiological surveillance and educational activities, and low articulation of partnerships with social actors and institutions [38–41].

The National Program for Improving Access and Quality of Primary Health Care (*Programa para Melhoria do Acesso e da Qualidade da Atenção Básica* [PMAQ]) is a program that encourages managers and teams to improve the quality of health services using a set of strategies for qualification, monitoring, and evaluation of the work of the health teams, including the transfer of resources. In 2012, this program revealed that 76.3% of the population was covered by primary healthcare teams and approximately 60% by teams of the Family Health Strategy (*Estratégia de Saúde da Família* [ESF]). The authors believe that, in the Northeast region in particular, the ESF has contributed to reduced inequalities in health. Despite promising coverage, some states, including those belonging to well-evaluated regions, had indicators of poor infrastructure (Mato Grosso, Rondônia, Pará, Distrito Federal, Rio de Janeiro, and São Paulo) [75]. It is noteworthy that only 64% of the primary healthcare centers investigated by PMAQ had a computer, and of these, only 45% had internet access. The North and the Northeast regions had the lowest percentages of adequate healthcare infrastructure, 42% and 22%, respectively. Of the municipalities in this region, the smaller ones had the least access (56%) to household follow-up leprosy records, available in the Basic Attention Information System [76]. Finally, the PMAQ also pointed out that the availability of drugs in the primary healthcare centers evaluated (about 38,000) was positively associated with the provision of better organized pharmaceutical services and the presence of ESF teams [77].

This scenario shows that although social determinants of leprosy influence the incidence of cases, Brazil has important weaknesses in the organization of services, particularly in primary healthcare. This leads to inadequacy in the prevention and diagnosis of the disease and in the control of thalidomide use. It is clear that to reduce the burden of the disease on the population and to effectively control the distribution and use of thalidomide, there is a need for differentiated standards of health, infrastructure, and management teams. These teams must have a broader view of the health needs of the population and the ability to manage these services. There is also a need to prioritize improvements in the quality of services in the areas that are more endemic for leprosy (North, Northeast, and Central-West), which have a precarious public health service and also consume the largest amounts of thalidomide in Brazil. In a recent study conducted by researchers in Brazil, with socioeconomic data from the cohort of 100 million Brazilians, it was identified that “within the poorest half of the Brazilian population, the most deprived individuals have the greatest risk of leprosy. Strategies focusing on early detection and treatment in the poorest populations could contribute substantially to global disease control.” Furthermore, along with early diagnosis and treatment of leprosy cases, social development is a key strategy for reducing the prevalence of leprosy worldwide [78].

Another important aspect to note is the therapeutic benefits of thalidomide, mainly in cases of ENL. Thalidomide’s use as a therapeutic justified its return to the world market, on the condition that the main adverse effect resulting from its use would be avoided. The regulatory changes and the structuring of services were the main focus of the governments of several countries. However, this essentiality of the use of thalidomide suppressed the importance of the development of new technologies, which could replace thalidomide in the first-line treatment of ENL. Some studies have shown all currently available treatment modalities have some drawbacks and are not effective to control the leprosy reaction for all patients because most of them present ENL recurrently or chronically. Moreover, there are few quality controlled studies on ENL treatment, which were performed more than 20 years ago [25], making it difficult to provide safe and effective therapeutic alternatives to thalidomide.

As demonstrated by Santana and colleagues (2017), there is a lack of investment in the research of medicines to treat poverty-related diseases, a reflection of health inequities, of which leprosy is an example. The lack of interest by the pharmaceutical industry in this type of research has led to a low number of innovations and transfers to the state the responsibility of providing access to the best therapies. The authors highlight that an equitable alternative for analysis of technologies can be centered on the development by the SUS of activities known as Horizon Scanning (*Monitoramento do Horizonte Tecnológico* [MHT]) for poverty-related diseases. This strategy would contribute to forming a definition of priority technologies for the SUS and the orientation of investment in research and development of medicines that meet national needs [79].

## Conclusion

Despite the advances in the health surveillance public policies in Brazil, the rational use of thalidomide by the population is still a challenge. The Brazilian scenario is a complex one, and it is in such a trying scenario that the control of thalidomide use is inserted. Even though the MoH and the ANVISA have promoted initiatives to ensure patients’ safety and minimize exposure to risks, such as training programs, these actions have not been enough. First, the majority of patients who make use of this medicine are in vulnerable socioeconomic conditions, which prevent adherence to treatment. Secondly, the precarious organization and structure of the primary healthcare services, particularly in remote areas, are impediments to an effective commitment of the health professionals and managers.

Health actions must integrate educational campaigns for the general public, not excluding popular mobilization, as well as continuous education for all health professionals in every level of care. Such educational actions should emphasize the precautions and risks inherent to the treatment to ensure high-quality standards. There is also the need for allotting resources to improve the computerized monitoring system not only for the control of the medicine use but also the congenital defects vigilance.

The identification of cases of pregnant women using thalidomide in Brazil suggests that the country needs to redesign the thalidomide access flow, including medical care in specialized health centers, which have high-quality structure and pharmaceutical services permanently available.

The government's actions in fostering and developing technologies in the SUS are key to defining new lines of treatment for ENH. Parallel to that, it is also possible to minimize the exposure risks to thalidomide embryopathy by instituting a PCDT for the treatment of ENH that allows the direction and unification of the use of thalidomide for this clinical condition, to establish criteria for treatment, also considering other already available drugs such as prednisone and pentoxifylline. In addition, the Brazilian health system must prioritize the development of research that makes it possible to identify safe and effective therapeutic alternatives to thalidomide. This measure allows the establishment of a deadline for thalidomide to be replaced with safer and effective treatment alternatives.

Existing studies highlight the fundamental role of public policies for reducing the prevalence of leprosy and reaching the goals of the WHO Global Leprosy Strategy 2016–2020. Interrupting the transmission of the disease means investing efforts in increasing diagnosis, early detection and treatment, as well as improving access to healthcare and conditions of subsistence for the poorest populations in endemic regions. Brazil must set a goal of eliminating leprosy in the country because it has not yet reached the target set by WHO.

Finally, it is important the development of other studies that address the control of thalidomide use in countries endemic for leprosy and also develop a comparative table of the control system in various countries.

### Key learning points

1. There are still cases of pregnant women using thalidomide in Brazil.
2. Brazil has a high consumption rate of thalidomide associated with the endemicity of leprosy.
3. Brazil’s organization and structure of primary healthcare impact the control of thalidomide use.
4. There are no safe substitutes to thalidomide available in Brazil's Unified Health System for erythema nodosum leprosum.
5. It is critical to implement quality clinical protocols for the safe use of thalidomide in erythema nodosum leprosum.

### Top five papers

1. Nery JS, Ramond A, Pescarini JM, Alves A, Strina A, Ichihara MY et al. Socioeconomic determinants of leprosy new case detection in the 100 Million Brazilian Cohort: a population-based linkage study. Lancet Global Health. 2019;7(9):PE1226-E1236. https://doi.org/10.1016/S2214-109X(19)30260-8 PMID: 31331811
2. Costa PDSS, Fraga LR, Kowalski TW, Daxbacher ELR, Schuler-Faccini L, Vianna FSL. Erythema Nodosum Leprosum: Update and challenges on the treatment of a neglected condition. Acta Trop. 2018;183:134–141. https://doi.org/10.1016/j.actatropica.2018.02.026 PMID 29474830
3. Santana RS, de Oliveira Lupatini E, Zanghelini F, de March Ronsoni R, Rech N, Leite SN. The different clinical guideline standards in Brazil: High cost treatment diseases versus poverty-related diseases. PLoS ONE. 2018;13(10):e0204723. http://doi.org/10.1371/journal.pone.0204723 PMID: 30332422
4. Vianna FSL, Lopez-Camelo JS, Leite JC, Sanseverino MT, Dutra Mda G, Castilla EE, et al. Epidemiological Surveillance of Birth Defects Compatible with Thalidomide Embryopathy in Brazil. PLoS ONE. 2011;6(7):e21735. https://doi.org/10.1371/journal.pone.002173 PMID 21754997
5. Vianna FSL, Oliveira MZ, Sanseverino MT, Morelo EF, de Lyra Rabello Neto D, Lopez-Camelo J, et al. Pharmacoepidemiology and thalidomide embryopathy surveillance in Brazil. Reprod Toxicol. 2015;53:63–67. https://doi.org/10.1016/j.reprotox.2015.03.007 PMID 25828060

## Supporting information

S1 Table Number of 100 mg thalidomide tablets acquired by the Brazilian Ministry of Health for the treatment of ENL and other clinical conditions between 1998 and 2018. Other clinical conditions (lupus, multiple myeloma, aphthous ulcers in patients with HIV-AIDS, graft-versus-host disease, and myelodysplastic syndrome). ENL, erythema nodosum leprosum.
(DOCX)

S2 Table Distribution of 100 mg thalidomide tablet by the Brazilian Ministry of Health for the treatment of ENL and other clinical conditions between 1998 and 2018. Other clinical conditions (lupus, multiple myeloma, aphthous ulcers in patients with HIV-AIDS, graft-versus-host disease, and myelodysplastic syndrome). ENL, erythema nodosum leprosum.
(DOCX)

S1 Appendix Rate of distribution of 100 mg thalidomide tablet. (DOCX)
