# Supplementary material for: The organization, weaknesses, and challenges of the control of thalidomide in Brazil: A review
Source: PLoS Negl Trop Dis. 2020 Aug 6;14(8):e0008329. doi: 10.1371/journal.pntd.0008329 (PMC7410199; doi:10.1371/journal.pntd.0008329)
Supplement: S1 Table — Other clinical conditions (lupus, multiple myeloma, aphthous ulcers in patients with HIV-AIDS, graft-versus-host disease, and myelodysplastic syndrome). ENL, erythema nodosum leprosum. (DOCX) [file pntd.0008329.s001.docx]

S1 Table. Number of 100 mg thalidomide tablet acquired by the Brazilian Ministry of Health for the treatment of ENL and other clinical conditions between 1998 and 2018.

| **Year** | **Contract number** | **Public laboratory** | **Quantity total** | **ENL** | **Other clinical conditions** |
| --- | --- | --- | --- | --- | --- |
| **1998** | 02/1998 | Funed | 4,908,480 | 3,801,600 | 1,106,880 |
| **1999** | 31/1999 | Funed | 2,535,360 | 2,535,360 | - |
| **2000** | 81/1999 | Funed | 2,809,920 | 2,809,920 | - |
| **2001** | 13/2001 | Funed | 46,560 | - | 46,560 |
| **2001** | 27/2001 | Funed | 3,142,080 | 3,142,080 | - |
| **2001** | 57/2001 | Funed | 2,100,000 | 592,800 | 1,507,200 |
| **2002** | 34/2002 | Funed | 3,442,560 | 3,211,200 | 231,360 |
| **2002** | 35/2002 | Funed | 46,560 | - | 46,560 |
| **2003** | 18/2003 | Funed | 240,000 | - | 240,000 |
| **2003** | 17/2003 | Funed | 1,726,560 | 1,726,560 | - |
| **2003** | 61/2003 | Funed | 1,004,160 | 1,004,160 | - |
| **2003** | 76/2003 | Funed | 840,480 | - | 840,480 |
| **2004** | 14/2004 | Funed | 60,000 | - | 60,000 |
| **2004** | 31/2004 | Funed | 6,091,680 | 4,455,840 | 1,635,840 |
| **2005** | 02/2005 | Funed | 6,288,960 | 4,444,800 | 1,844,160 |
| **2005** | 02/2005 | Funed | 48,000 | - | 48,000 |
| **2006** | 01/2006 | Funed | 76,800 | - | 76,800 |
| **2006** | 03/2006 | Funed | 5,756,160 | 3,754,560 | 2,001,600 |
| **2006** | 19/2006 | Funed | 3,133,920 | 3,133,920 | - |
| **2006** | 20/2006 | Funed | 120,000 | - | 120,000 |
| **2007** | 03/2007 | Funed | 1,896,480 | - | 1,896,480 |
| **2007** | 09/2007 | Funed | 96,000 | - | 96,000 |
| **2008** | 02/2008 | Funed | 6,207,840 | 3,740,640 | 2,467,200 |
| **2009** | 05/2009 | Funed | 6,850,560 | 4,199,040 | 2,651,520 |
| **2010** | 07/2010 | Funed | 6,154,080 | 3,512,640 | 2,641,440 |
| **2011** | 02/2011 | Funed | 3,079,200 | 1,338,720 | 1,740,480 |
| **2012** | 16/2012 | Funed | 3,209,760 | 2,164,800 | 1,044,960 |
| **2013** | 1220/13 | Funed | 7,238,880 | 4,856,160 | 2,382,720 |
| **2014** | 103/2014 | Funed | 7,452,000 | 5,669,760 | 1,782,240 |
| **2016** | 64/2016 | Funed | 6,334,560 | 4,068,000 | 2,266,560 |
| **2017** | 135/2017 | Funed | 5,433,600 | 4,401,600 | 1,032,000 |
| **2018** | 244/2018 | Funed | 5,518,080 | 4,192,800 | 1,325,280 |
| **Total** | **-** | **-** | **103,889,280** | **72,756,960** | **31,132,320** |

Erythema Nodosum Leprosum (ENL). Other clinical conditions (lupus, multiple myeloma, aphthous ulcers in patients with HIV-AIDS, graft-versus-host disease and myelodysplastic syndrome). Ezequiel Dias Foundation (*Fundação Ezequiel Dias-*FUNED).
