# Supplementary material for: The organization, weaknesses, and challenges of the control of thalidomide in Brazil: A review
Source: PLoS Negl Trop Dis. 2020 Aug 6;14(8):e0008329. doi: 10.1371/journal.pntd.0008329 (PMC7410199; doi:10.1371/journal.pntd.0008329)
Supplement: S2 Table — Other clinical conditions (lupus, multiple myeloma, aphthous ulcers in patients with HIV-AIDS, graft-versus-host disease, and myelodysplastic syndrome). ENL, erythema nodosum leprosum. (DOCX) [file pntd.0008329.s002.docx]

| **S2 Table. Distribution of 100 mg thalidomide tablet by the Brazilian Ministry of Health for the treatment of ENL and other clinical conditions between 1998** | | | | | | | | | | | | |  |  |
| --- | --- | --- | --- | --- | --- | --- | --- | --- | --- | --- | --- | --- | --- | --- |
| **and 2018.** | | | | | | | | | | | |  |  |  |
| **States** | **2005** | | | **2006** | | | **2007** | | | **2008** | | | | |
|  | **ENL** | **Other clinical conditions** | **HIV-AIDS** | **ENL** | **Other clinical conditions** | **HIV-AIDS** | **ENL** | **Other clinical conditions** | **HIV-AIDS** | **ENL** | **Other clinical conditions** | | | **HIV-AIDS** |
| **Acre** |  |  |  |  | 4,320 |  |  |  |  | 11,040 |  | | |  |
| **Alagoas** |  |  | 480 | 21,600 |  |  | 9,600 |  | 480 | 10,050 | 10,560 | | | 480 |
| **Amazonas** | 11,040 |  |  |  | 4,800 | 480 |  | 32,160 | 2,880 | 4,800 |  | | | 2,400 |
| **Amapá** | 5,280 |  |  |  |  |  |  |  |  | 2,400 | 6,720 | | | 960 |
| **Bahia** |  |  |  |  | 26,880 | 480 | 126,240 |  | 480 |  |  | | |  |
| **Ceará** | 72,000 |  | 960 |  | 37,440 |  | 39,360 |  | 1,920 | 107,040 |  | | | 960 |
| **Distrito Federal** | 106,560 |  | 960 | 6,240 | 27,840 | 5,760 |  | 6,720 | 5,760 | 112,320 |  | | | 7,200 |
| **Espírito Santo** | 10,080 |  |  |  | 51,360 |  |  |  | 480 | 58,080 | 19,680 | | |  |
| **Goiás** |  |  |  |  | 14,880 |  |  |  |  | 19,680 |  | | |  |
| **Maranhão** | 54,240 |  |  |  |  |  |  |  | 4,320 | 33,600 |  | | | 1,920 |
| **Mato Grosso** | 32,160 |  | 960 | 31,680 |  |  |  | 13,920 | 960 | 146,880 |  | | | 4,800 |
| **Mato Grosso do Sul** | 10,080 |  |  |  |  |  |  | 20,160 |  | 12,000 |  | | | 960 |
| **Minas Gerais** | 39,360 |  |  | 49,920 | 87,360 | 480 | 24,960 | 105,600 | 480 | 79,680 |  | | | 2,400 |
| **Pará** | 55,200 |  |  |  | 23,040 |  |  |  |  | 27,840 |  | | |  |
| **Paraíba** | 32,160 |  |  | 16,320 |  |  |  |  |  | 11,040 |  | | |  |
| **Paraná** |  |  | 1,440 |  | 33,600 | 1,440 |  | 58,080 | 960 |  |  | | |  |
| **Pernambuco** | 74,400 |  |  |  | 10,080 | 8,640 | 18,240 | 37,440 |  |  | 36,000 | | |  |
| **Piauí** |  |  |  |  |  |  |  |  |  | 54,240 |  | | |  |
| **Rio de Janeiro** |  |  | 1,920 | 11,520 | 40,800 | 4,800 | 124,800 | 480 | 7,200 | 101,280 | 40,320 | | | 5,760 |
| **Rio Grande do Norte** | 21,600 |  | 480 | 17,280 | 5,280 | 480 |  | 20,160 |  | 25,920 |  | | | 4,800 |
| **Rio Grande do Sul** |  |  |  |  | 46,560 |  |  | 960 | 480 |  |  | | | 2,400 |
| **Rondônia** | 18,240 |  |  | 49,440 |  |  | 50,400 |  |  |  |  | | |  |
| **Roraira** |  |  |  |  |  |  |  |  |  | 2,880 |  | | |  |
| **Santa Catarina** | 12,960 |  |  |  |  |  | 25,440 | 36,000 | 480 | 9,600 |  | | | 480 |
| **São Paulo** | 5,280 |  | 8,160 |  | 157,920 | 5,760 | 354,720 | 269,760 | 17,280 | 30,240 | 49,920 | | | 26,400 |
| **Sergipe** | 111,360 |  |  |  |  |  | 21,120 |  |  | 8,640 | 11,040 | | |  |
| **Tocantins** |  |  |  |  |  |  |  | 6,240 |  | 20,160 | 12,000 | | |  |
| **Total** | **672,000** | **0** | **15,360** | **204,000** | **572,160** | **28,320** | **794,880** | **607,680** | **44,160** | **889,410** | **186,240** | | | **61,920** |

Erythema Nodosum Leprosum (ENL). Other clinical conditions (lupus, multiple myeloma, aphthous ulcers in patients with HIV-AIDS, graft-versus-host disease and myelodysplastic syndrome).

| **2009** | | | **2010** | | | **2011** | | | **2012** | | |
| --- | --- | --- | --- | --- | --- | --- | --- | --- | --- | --- | --- |
| **ENL** | **Other clinical conditions** | **HIV-AIDS** | **ENL** | **Other clinical conditions** | **HIV-AIDS** | **ENL** | **Other clinical conditions** | **HIV-AIDS** | **ENL** | **Other clinical conditions** | **HIV-AIDS** |
| 6,720 |  |  |  |  |  | 6,720 |  |  | 5,760 | 38,880 |  |
| 7,680 |  |  | 15,360 |  |  | 21,600 |  |  | 5,280 | 35,520 |  |
| 20,640 |  | 960 |  |  |  | 20,160 |  | 3,360 | 5,760 | 4,320 |  |
| 6,240 |  |  |  |  |  | 13,440 |  |  | 2,400 | 18,240 |  |
|  |  | 1,440 | 18,240 |  |  | 184,320 |  |  | 49,920 | 285,600 | 1,020 |
| 40,320 |  | 480 | 960 |  | 480 | 84,000 |  | 4,110 | 24,000 | 123,360 | 3,240 |
| 26,880 |  |  |  |  |  | 18,720 |  |  | 7,680 | 47,850 |  |
| 54,720 |  | 480 |  | 9,600 |  | 157,920 |  |  | 48,000 | 62,400 | 570 |
| 99,840 | 11,520 |  |  | 30,240 |  | 254,400 |  |  |  | 151,800 |  |
| 48,960 |  | 960 | 1,650 |  | 480 | 109,440 | 480 | 2,580 | 19,200 | 22,080 | 1,470 |
| 51,960 |  | 480 |  |  |  | 135,840 |  | 780 | 24,000 | 157,440 | 1,890 |
| 28,800 |  |  | 17,280 |  |  | 55,290 | 21,120 | 870 | 10,560 | 22,080 |  |
| 52,320 | 40,320 | 1,920 | 480 | 138,240 | 480 |  |  |  | 90,240 | 210,780 | 180 |
|  |  | 480 |  |  |  | 161,280 |  |  | 47,040 | 70,080 |  |
| 16,320 |  |  | 42,240 |  |  | 89,760 |  |  | 42,240 |  |  |
|  |  | 960 | 148,800 | 99,840 |  | 148,320 |  |  | 4,800 | 116,160 | 840 |
| 60,000 |  |  | 960 | 75,360 |  | 102,720 |  | 480 | 88,800 | 147,840 | 510 |
| 21,600 | 9,600 |  | 24,000 |  | 15,360 | 109,440 |  |  |  | 36,480 | 600 |
| 38,880 | 480 | 1,920 | 1,920 | 96,000 | 2,400 | 37,920 |  | 6,750 | 6,240 | 1,080 | 6,780 |
| 8,160 |  |  |  |  |  | 24,000 |  |  | 20,160 | 46,080 |  |
| 8,160 |  | 960 | 1,680 |  | 480 |  |  | 480 |  |  |  |
| 75,360 |  |  |  |  |  | 7,680 |  |  | 32,640 | 44,160 |  |
| 27,360 |  |  |  |  |  | 7,200 |  |  | 3,840 | 7,200 |  |
| 25,920 |  | 960 |  |  | 960 | 4,800 |  | 1,080 | 28,320 | 49,920 | 1,230 |
| 50,400 | 50,880 | 40,800 | 7,800 |  | 4,800 | 190,560 | 100,320 | 10,800 | 40,320 | 223,800 | 6,720 |
| 12,480 |  |  | 3,360 |  |  | 25,440 |  |  | 12,000 | 23,040 |  |
| 24,960 |  |  |  | 14,400 |  | 18,240 |  |  | 17,760 | 24,960 |  |
| **814,680** | **112,800** | **52,800** | **284,730** | **463,680** | **25,440** | **1,989,210** | **121,920** | **31,290** | **636,960** | **1,971,150** | **25,050** |

| **2013** | | | **2014** | | | **2015** | | | **2016** | | |
| --- | --- | --- | --- | --- | --- | --- | --- | --- | --- | --- | --- |
| **ENL** | **Other clinical conditions** | **HIV-AIDS** | **ENL** | **Other clinical conditions** | **HIV-AIDS** | **ENL** | **Other clinical conditions** | **HIV-AIDS** | **ENL** | **Other clinical conditions** | **HIV-AIDS** |
| 1,920 | 480 |  | 5,760 | 11,520 |  | 6,800 | 2,880 |  | 20,160 | 960 |  |
| 31,200 | 1,440 |  | 43,200 |  |  | 46,080 | 5,280 |  | 41,880 |  |  |
| 11,520 |  |  | 69,600 | 12,960 |  | 42,720 | 2,400 |  | 53,280 | 11,040 |  |
| 5,280 |  |  | 5,760 | 8,160 |  | 6,240 |  |  | 7,680 | 960 |  |
| 153,600 | 480 |  | 564,480 | 17,280 |  | 211,680 | 28,800 | 480 | 316,160 |  |  |
| 71,520 | 12,000 | 1,590 | 41,280 | 12,480 |  | 93,600 | 7,200 | 960 | 185,760 | 25,920 | 480 |
| 24,480 | 24,000 |  | 82,080 | 60,000 |  | 54,720 | 23,520 |  | 36,480 | 36,000 |  |
| 44,640 | 2,400 | 60 | 57,600 | 39,840 |  | 66,240 |  | 960 | 54,240 | 16,800 |  |
|  |  |  | 460,800 | 74,880 |  | 216,090 | 34,080 | 480 | 211,200 | 37,920 |  |
| 84,480 | 3,360 | 300 | 250,080 | 11,040 |  | 175,200 | 8,640 | 10,080 | 201,120 | 6,240 |  |
| 75,840 | 4,800 | 300 | 144,960 | 10,560 |  | 82,560 | 3,840 | 3,360 | 80,640 | 2,400 |  |
| 25,440 | 18,720 |  | 102,720 | 14,400 |  | 23,040 | 8,160 |  | 63,840 | 24,000 |  |
|  |  |  | 271,200 | 128,160 |  | 180,960 | 163,680 | 480 | 199,680 | 89,280 | 480 |
| 156,960 | 20,080 |  | 327,840 | 21,600 |  | 138,240 | 6,240 |  | 182,400 | 2,400 |  |
| 10,560 | 1,920 |  | 70,080 | 22,080 |  | 41,280 | 21,120 |  | 65,280 | 4,320 |  |
| 85,920 | 24,480 | 90 | 248,160 | 54,240 |  | 146,400 | 38,880 | 480 | 178,560 | 36,480 |  |
| 60,960 | 33,120 | 510 | 212,160 | 46,560 |  | 90,240 | 18,240 |  | 142,560 | 26,880 |  |
| 27,360 | 5,280 |  | 141,600 | 17,760 |  | 57,600 | 2,400 |  | 65,280 | 5,280 | 480 |
| 73,920 | 78,720 | 6,120 | 231,360 | 210,240 |  | 45,600 | 72,960 | 8,640 | 88,320 | 73,440 | 780 |
| 4,800 | 7,200 |  | 72,480 | 9,120 |  | 27,360 | 26,880 |  | 17,760 | 36,000 |  |
| 27,360 | 24,000 |  | 25,920 | 75,840 |  | 30,720 | 61,440 |  | 29,760 | 54,720 |  |
| 22,560 | 28,230 |  | 46,560 |  |  | 27,840 |  |  | 82,080 |  |  |
| 3,840 | 4,320 |  | 4,320 |  |  | 9,120 |  |  | 12,480 | 960 |  |
| 26,880 | 12,960 | 120 | 46,560 | 61,440 |  | 32,160 | 37,440 |  | 38,400 | 27,840 |  |
| 299,040 | 50,400 | 6,330 | 419,040 | 534,240 |  | 103,200 | 165,120 |  | 189,120 | 204,960 | 1,920 |
|  |  |  | 21,120 | 48,880 |  | 1,440 | 45,600 |  | 20,160 | 2,880 |  |
| 10,560 |  |  | 78,240 | 3,360 |  | 61,440 | 5,760 | 480 | 55,680 | 2,400 |  |
| **1,340,640** | **358,390** | **15,420** | **4,044,960** | **1,506,640** | **0** | **2,018,570** | **790,560** | **26,400** | **2,639,960** | **730,080** | **4,140** |

| **2017** | | | **2018** | | | **Total** | | |
| --- | --- | --- | --- | --- | --- | --- | --- | --- |
| **ENL** | **Other clinical conditions** | **HIV-AIDS** | **ENL** | **Other clinical conditions** | **HIV-AIDS** | **ENL** | **Other clinical conditions** | **HIV-AIDS** |
| 21,120 | 5,760 |  | 8,640 | 1,440 |  | 94,640 | 66,240 | 0 |
| 105,120 |  |  | 27,840 |  |  | 386,490 | 52,800 | 1,440 |
| 83,040 | 28,800 |  | 21,780 | 3,840 |  | 344,340 | 100,320 | 10,080 |
| 11,040 | 3,840 |  | 7,200 | 960 |  | 72,960 | 38,880 | 960 |
| 528,960 |  |  | 238,560 | 960 |  | 2,392,160 | 360,000 | 3,900 |
| 234,720 | 62,880 |  | 64,320 | 55,680 |  | 1,058,880 | 336,960 | 15,180 |
| 29,760 | 97,920 |  | 22,080 |  |  | 528,000 | 323,850 | 19,680 |
| 93,120 | 43,200 |  | 27,840 | 7,680 |  | 672,480 | 252,960 | 2,550 |
| 334,560 | 80,640 |  | 158,880 | 24,000 |  | 1,755,450 | 459,960 | 480 |
| 288,480 | 10,080 |  | 134,880 | 1,440 |  | 1,401,330 | 63,360 | 22,110 |
| 190,560 | 13,440 |  | 76,320 |  |  | 1,073,400 | 206,400 | 13,530 |
| 88,800 | 40,320 |  | 36,960 | 960 |  | 474,810 | 169,920 | 1,830 |
| 351,360 | 288,000 | 960 | 45,120 | 137,800 |  | 1,385,280 | 1,389,220 | 7,860 |
| 364,320 |  |  | 120,960 | 7,200 |  | 1,582,080 | 150,640 | 480 |
| 76,320 | 41,280 |  | 27,840 | 17,760 |  | 541,440 | 108,480 | 0 |
| 361,920 | 70,080 |  | 92,640 | 21,120 |  | 1,415,520 | 552,960 | 6,210 |
| 248,160 | 100,800 |  | 28,800 | 12,480 |  | 1,128,000 | 544,800 | 10,140 |
| 132,000 | 13,920 |  | 51,840 | 1,920 |  | 684,960 | 92,640 | 16,440 |
| 194,400 | 144,960 |  | 81,600 | 36,960 |  | 1,037,760 | 796,440 | 53,070 |
| 24,000 | 60,480 | 960 | 24,480 | 10,560 |  | 288,000 | 221,760 | 6,720 |
| 44,640 | 85,920 |  | 8,640 | 32,160 |  | 176,880 | 381,600 | 4,800 |
| 125,760 |  |  | 51,840 |  |  | 590,400 | 72,390 | 0 |
| 17,760 | 3,360 |  | 7,680 |  |  | 96,480 | 15,840 | 0 |
| 71,520 | 68,160 |  | 27,360 | 26,400 |  | 349,920 | 320,160 | 5,310 |
| 493,440 | 716,640 |  | 246,240 | 241,440 |  | 2,429,400 | 2,765,400 | 128,970 |
| 44,640 | 16,800 | 3,360 | 29,280 |  |  | 311,040 | 148,240 | 3,360 |
| 71,040 |  |  | 33,600 |  |  | 391,680 | 69,120 | 480 |
| **4,630,560** | **1,997,280** | **5,280** | **1,703,220** | **642,760** | **0** | **22,663,780** | **10,061,340** | **335,580** |
