# Supplementary material for: The organization, weaknesses, and challenges of the control of thalidomide in Brazil: A review
Source: PLoS Negl Trop Dis. 2020 Aug 6;14(8):e0008329. doi: 10.1371/journal.pntd.0008329 (PMC7410199; doi:10.1371/journal.pntd.0008329)
Supplement: S1 Appendix — (DOCX) [file pntd.0008329.s003.docx]

**S1 Appendix. Rate of distribution of 100 mg thalidomide tablet**

**Use**

Measure the number of tablets delivered.

**Interpretation**


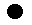
 < 100.00 tablets/10,000 inhabitants


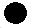


101.00 to 200.00 tablets/10,000 inhabitants


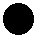


201.00 to 300.00 tablets/10,000 inhabitants


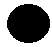


> 301.00 tablets/10,000 inhabitants

**Calculation method**

Numerator: Number of 100 mg thalidomide tablets in the state until 12/31 of the evaluation year

Denominator: total population in the state until 12/31 of the evaluation year

Multiplication factor: 10,000
